# Does social distance modulate adults’ egocentric biases when reasoning about false beliefs?

**DOI:** 10.1371/journal.pone.0198616

**Published:** 2018-06-08

**Authors:** Benjamin G. Farrar, Ljerka Ostojić

**Affiliations:** Department of Psychology, University of Cambridge, Cambridge, United Kingdom; TNO, NETHERLANDS

## Abstract

When given privileged information of an object’s true location, adults often overestimate the likelihood that a protagonist holding a false belief will search in the correct location for that object. This type of egocentric bias is often labelled the ‘curse of knowledge’. Interestingly, the magnitude of this bias may be modulated by the social distance between the perspective taker and target. However, this social distance effect has yet to be fully demonstrated when adults reason about false beliefs. Using a continuous false belief task, we investigated i) whether adults were biased by their own knowledge when reasoning about another’s false belief, ii) whether the magnitude of this egocentric bias was modulated by social distance, and iii) whether this social distance effect extended to a heterospecific out-group, namely a dog. To test these hypotheses we conducted three experiments. In Experiment 1 (N = 283), we used an established continuous false belief task, in Experiment 2 (N = 281) we modified this task, and Experiment 3 (N = 744) was a direct replication of Experiment 2. Across these experiments, the curse of knowledge effect was reliably replicated when adults mentalised about an in-group protagonist, and replicated in two of the three studies (Experiments 1 and 3) when adults mentalised about out-group protagonists. In an internal-meta analysis, the curse of knowledge effect was present across all conditions, and there was no effect of social distance. Hence, overall these data are not consistent with the hypothesis that social distance modulates adults’ egocentric biases when reasoning about false beliefs. The finding that egocentric biases of a similar magnitude were observed when adults mentalised about an in-group protagonist and a dog suggests that interpersonal dissimilarity is not in itself sufficient to reduce egocentric bias when reasoning about false beliefs.

## Introduction

Although we possess the ability to attribute mental states to others, this so-called Theory of Mind is often sub-optimal. Both adults and children frequently over-extend their own mental states onto others. Such an egocentric bias is seen when we reason about what others see [1], feel [2], know [3], and believe [4].

Epley et al. [5] proposed that egocentric biases arise because perspective taking, including Theory of Mind, follows an anchor and adjustment mechanism (see also [6]). By this account, the perspective taker first projects their own mental state onto another, and then serially adjusts away from this egocentric anchor to arrive at the other’s perspective. When such adjustment is insufficient, the perspective taker is biased by their own perspective. For example, egocentric biases are observed when adults complete false belief tasks which measure a continuous dependent variable [4,7]. In their seminal study, Birch & Bloom [4] used a modified false belief task in which a protagonist, Vicki, placed her violin in a blue container, one of a total of four differently coloured containers. While Vicki was away, her sister moved the violin to either i) “another container”, or ii) “the red container”, and then switched the locations of all containers. Next, participants rated the percentage likelihood that Vicki, upon return, would search for her violin in each of the four containers. Despite Vicki’s belief of the violin’s location being identical between conditions, participants who knew the violin was in the red container reported it more likely that Vicki would search there, compared to participants ignorant of the violin’s true location.

Such a *curse of knowledge effect* in false belief reasoning has been replicated both conceptually, by using a different paradigm [7], and by four additional studies using the Birch & Bloom paradigm [8–11]. However, Ryskin and Brown-Schmidt [12] recently reported a large-scale replication failure of the curse of knowledge effect. Across seven replicates of the Birch and Bloom paradigm [4], Ryskin and Brown-Schmidt reported a significant curse of knowledge effect in only three. Combining the effect size across all seven studies yielded a Cohen’s *d* of 0.20, considerably smaller than the 0.469 estimated from Birch and Bloom’s data.

However, the smaller effect size reported by Ryskin and Brown-Schmidt [12] could be explained by considering the social distance between the participants and protagonist in their study. Here, social distance is used as an umbrella term that incorporates several related factors, such as similarity, likeability, familiarity and group-membership, all of which may influence egocentricity during social inferences [13–15]. In two of Ryskin and Brown-Schmidt’s seven experiments, the protagonist Vicki was white with an English name and in the other five, as per Birch and Bloom’s original study, Vicki was East-Asian with an English name. As their sample was ethnically heterogeneous, Vicki likely represented an out-group for some of the participants (reanalysis of data made available by Ryskin, Oct 2016; now available from https://osf.io/kfskv/). This is an important consideration because social distance may modulate the magnitude of egocentric biases in perspective taking. For example, US adults only overextend their own visceral states such as cold and thirst onto similar others [16] and students at American universities suffer greater egocentric intrusion when taking the visual perspective of an in-group rather than an out-group member [17]. Even young children find it easier to predict the future preferences of socially distant as opposed to socially close others [15]. Results such as these reinforce Tamir and Mitchell’s [6] suggestion that the egocentric anchoring and adjustment mechanism might be specific to mentalising about similar others, and that self-knowledge might not be recruited when mentalising about dissimilar others. Consistent with this hypothesis, Todd et al. ([11]: Experiment 4) demonstrated that adults displayed no egocentric bias when reasoning about out-group members’ false beliefs (*social distance effect*). Using the Birch and Bloom paradigm, Todd et al. replicated the curse of knowledge effect when German participants mentalised about a German in-group protagonist, Vicki, but not when they mentalised about a Turkish out-group protagonist, Yesim. Furthermore, Sassenrath et al. ([10]: Experiment 2) showed that participants who held a cup of cold water displayed smaller egocentric biases than participants who held a warm cup of water, again using the Birch and Bloom paradigm. As physical cold is associated with social distance [18], this finding supports the hypothesis that social distance modulates egocentric biases in false belief reasoning.

Although Todd et al. [11] and Sassenrath et al. [10] presented data numerically in line with a social distance effect, their critical statistical tests used both participants’ ratings that Vicki would look in the “red container” and that she would look in the “blue container” as simultaneous dependent variables. However, because in the Birch and Bloom paradigm the ratings assigned to each container are non-independent, these analyses violated the key independence assumption of Analysis of Variance (ANOVA) [12,19]. However, a re-analysis of Todd et al.’s data (performed by Andrew Todd, personal communication, and independently performed by us), using just the ratings for the critical red container [8,12] did reveal a significant Location Knowledge x Protagonist interaction, *F*(1, 112) = 4.65, *p* = .033, η^2^ = .04, confirming the presence of a social distance effect.

However, the generalisability of Todd et al.’s results [11] may be limited by their use of only a single out-group manipulation. Moreover, the strength of this Turkish out-group protagonist may have varied between the German participants, for example as a function of how much intergroup contact they had experienced [20,21]. As the social distance effect *in general* could explain Ryskin and Brown-Schmidt’s replication failure [12] of the original Birch and Bloom study [4], it is necessary to attempt to replicate the curse of knowledge and social distance effects using a different, stronger and more consistent human out-group. A further method of creating a strong and consistent out-group may be possible by using non-human animals as protagonists. Eddy, Gallup and Povinelli [22] collected data on how American students perceived the similarity of a series of animals to themselves, and also the perceived higher order cognitive abilities of these animals, specifically those related to Theory of Mind. Following humans, the most cognitively able and similar animals were rated as primates, dogs and cats, in that order. The high cognitive rating of these non-human animals suggests adults will readily attribute beliefs to them. Crucially, adults also rated primates, dogs and cats as much more dissimilar to themselves than other humans, suggesting a large amount of perceived social distance between human adults and these non-human animals. This combination of perceived mental state attribution abilities and perceived dissimilarity indicates that these animals could act as a strong out-group to test the effects of social distance on egocentric biases in mental state attribution. Within these animals, the shared environments of dogs and cats with humans means ecologically valid paradigms can be designed that can allow the direct comparison of adult reasoning about human and non-human animal mental states.

The present study therefore had three aims: i) to test whether US and UK adults were biased by their own privileged knowledge when reasoning about another’s false belief, ii) to test whether the magnitude of this egocentric bias was modulated by social distance, and iii) to expand the study of social distance effects by including an ecological out-group, namely a dog, that would introduce a qualitative change in social distance. These aims were tested across three experiments. Experiment 1 used the Birch and Bloom modified false belief task with three protagonists: a white Western European in-group, a West African out-group, and a heterospecific dog. Experiment 2 further adapted the modified false belief task to control for the possibility that participants inferred that the dog could smell the location of the item hidden in Experiment 1, and Experiment 3 was a direct replication of Experiment 2. Across all three experiments, if biased by their own privileged knowledge, participants informed of the true location of an object should rate it more likely that the protagonist would search there, compared to participants ignorant of the object’s true location. Further, if this effect is modulated by social distance, participants should be less biased when reasoning about a conspecific out-group protagonist’s false belief. Finally, save for floor effects, participants should be less biased again when reasoning about a heterospecific protagonist’s false belief.

## Experiment 1

### Methods

#### Participants

Participants were recruited online via Prolific (http://www.prolific.ac) and were of white Western European descent, aged between 18 and 35, and either UK or US nationals. Three hundred participants (50% male) were recruited and randomly assigned to one of six conditions, in a 3 (Protagonist: in-group, out-group, heterospecific) x 2 (Knowledge: informed, ambiguous) between-subjects design. Of the 300 participants, three failed to meet the inclusion criteria (aged 18–35, white Western European descent, UK or US nationals), eight failed to complete the task and six failed the suspicion check, leading to a final sample size of 283. Informed consent was gained from each participant, who received monetary compensation for completing the task. This study was approved by the Psychology Ethics Committee of the University of Cambridge.

#### Procedure

**Fig 1:**
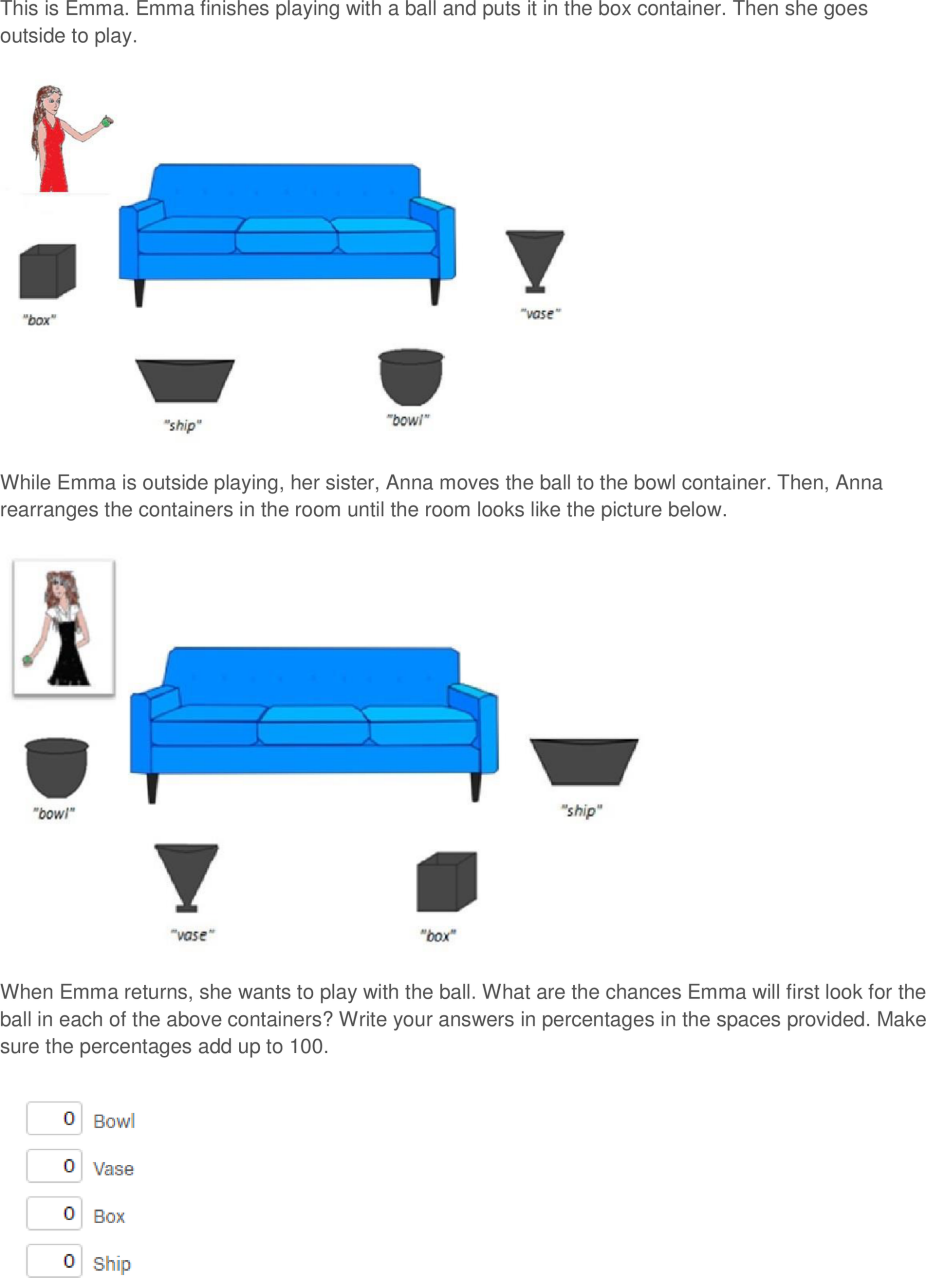
An example vignette of Experiment 1. The vignette presented to female participants in the in-group informed condition.

**Fig 2:**
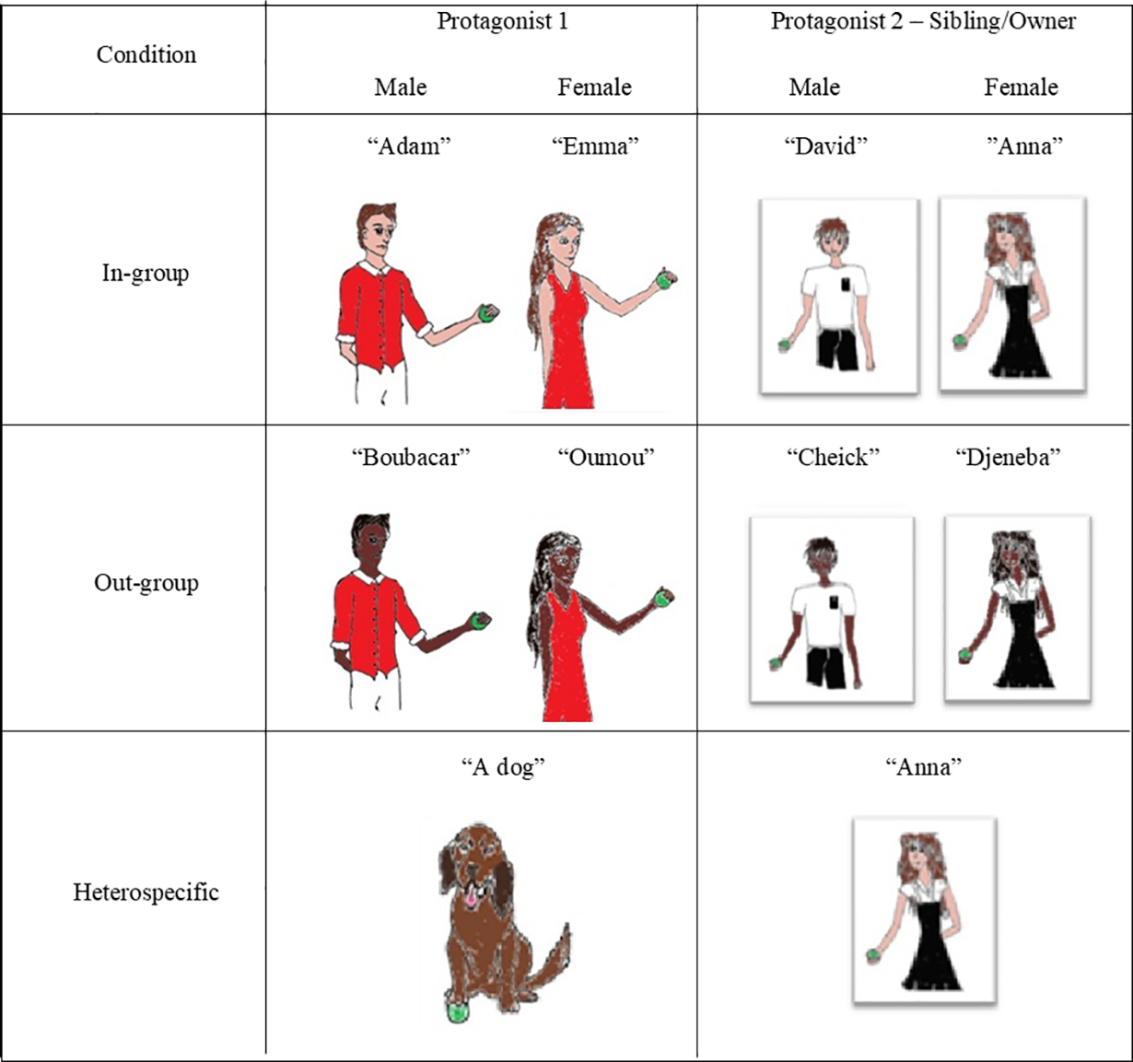
The protagonists used in each condition. Protagonists were gender-matched to participants, except in the heterospecific condition, where a dog and its female owner, Anna, were always presented.

Participants were presented the task online, hosted at Qualtrics (Qualtrics, Provo, UT, 2015), and completed an adaptation of a modified false belief task [4]. All participants were presented with a vignette consisting of two panels, involving two protagonists (see Fig 1 for an example vignette, and S1 and S2 Figs in the Supporting Information for examples of the other conditions). The first panel depicted a gender-matched protagonist playing with a ball (Protagonist 1), beside a sofa surrounded by four differently shaped containers. Instead of differently coloured containers [4], we used differently shaped containers to account for the differences in colour vision between humans and dogs. Following Todd et al. [11], the in-group and out-group conditions displayed the same images of the protagonists, with the skin and hair colour of the latter being darker. Images were hand drawn and edited using Microsoft Paint. Common names from each culture were given to each protagonist (see Fig 2 for the names and images of all protagonists). Aside from the protagonist, the stimuli were identical for all participants.

Participants first read about the protagonist placing a ball in one of the containers, e.g. “the box container”. Then, they were informed that while the protagonist was outside, their sibling/owner (Protagonist 2) moved the ball to either another, specific container, e.g. “the bowl container” (informed condition) or another, unspecified container, i.e. “another container” (ambiguous condition) and then switched the locations of all containers. Next, participants were asked to rate the percentage likelihood that the protagonist, upon return, would search for the ball in each of the four containers. Participants typed their answers in four labelled boxes (each indicating one of the differently shaped containers) below the panel and, following the procedure by Ryskin and Brown-Schmidt [12], the programme did not allow them to continue until their answers totalled 100. The order in which the boxes and the corresponding containers were presented was randomised between participants. Within each condition, the position of the relevant containers, and the location where the ball was first placed was counterbalanced across participants (see S1 Table in the Supporting Information for a full breakdown of the counterbalancing).

#### Analysis

Following Ryskin and Brown-Schmidt [12], to account for non-independence of the ratings for the different containes, we analysed the ratings for the container that was moved to the location where the protagonist originally put the ball, which was also the location where the ball was explicitly moved to in the informed condition (the bowl container in Fig 1 and henceforth the “true location container”). A Levene test was used to assess the homogeneity of variance of data between conditions, and–if appropriate–data were transformed using the procedure outlined by Warton and Hui [23] for the logit transformation of data including 0 and 1 values. For all analyses where logit transformed data were used, the corresponding analyses using untransformed data are presented in S1 Text in the Supporting Information. For all tests that were significant, or approached significance, the transformed data analyses provided more conservative results than the raw data analyses. Participants’ ratings of the true location container were submitted to a two-way analysis of variance (ANOVA), with Knowledge and Protagonist as between-subject factors. If, as predicted, participants were more biased by their own privileged knowledge in the in-group condition, rather than out-group or heterospecific conditions, a Knowledge x Protagonist interaction should show that the difference in participants’ ratings of the true location container between the informed and ambiguous conditions in the in-group condition was larger than in the out-group and heterospecific conditions. Post-hoc tests were conducted using independent samples t-tests with a Šidàk correction for multiple comparisons [24].

### Results

The variances of the true location container ratings were unequal across the different groups of participants (Levene test, *F*(5, 277) = 3.56, *p* = 0.004), thus data were logit transformed [23]. Transformed data were submitted to a two-way ANOVA, which yielded main effects of Knowledge, *F*(1, 277) = 11.51, *p* = 0.0008, η^2^ = 0.039, and Protagonist, *F*(2, 277) = 3.92, *p =* 0.021, η^2^ = 0.025 but no Knowledge x Protagonist interaction, (*F*(2, 277) = 0.635, *p* = 0.531, η^2^ = 0.005. The main effect of Knowledge was driven by participants in the informed condition giving higher ratings to the true location container (M = 38.72, SD = 26.61) than participants in the ambiguous condition (M = 26.93, SD = 19.62) (Table 1, ratings for all other containers are provided in S1 Text in the Supporting Information). Post-hoc analysis showed the main effect of Protagonist was driven by participants in the heterospecific condition giving overall higher ratings to the true location container than participants in the out-group condition (independent samples t-test, *t*(1, 187) = 2.73, *p*_*Šidàk*_ = 0.026, *d* = 0.397). Despite the absence of an interaction term, individual effect sizes for the curse of knowledge effect for each protagonist, using both the logit transformed data, and, in-line with previous studies, the raw data, were calculated to allow comparison with Birch and Bloom [4], *d* = 0.469, and Ryskin and Brown-Schmidt [12], *d* = 0.20.

**Table 1:** Mean (M) and standard deviation (SD) of participants’ ratings of the true location container in Experiment 1.

Knowledge	In-group	Protagonist Out-group	Heterospecific
Ambiguous	25.89 (21.76)	24.26 (15.90)	30.95 (20.68)
Informed ***	38.88 (26.72)	32.41 (27.47)	44.76 (24.68)
Curse of Knowledge Cohen’s *d—*logit	0.468	0.209	0.573
Curse of Knowledge Cohen’s *d—*raw	0.534	0.361	0.611

The “Knowledge” rows refer to the two manipulations of the participants’ own knowledge about the object’s final location was manipulated (“Ambiguous” and “Informed”). The “Protagonist” columns refer to the three manipulations of the social distance between the participants and the protagonist (“In-group”, “Out-group”, and “Heterospecific”). The last two rows give the effect sizes for the curse of knowledge effect (the difference between participants’ ratings in the ambiguous and informed conditions for each protagonist), which were calculated both using the logit transformed data used in the statistical analyses, and from the raw data, following [4] and [12].

*** denotes that the mean across the informed conditions differed significantly from the ambiguous conditions at α = 0.001.

The high ratings for the true location container in the heterospecific informed condition could, however, have been due to participants inferring the dog could smell the location of the ball, rather than being biased by their own private knowledge. For this reason, the heterospecific data were excluded and the conspecific data re-submitted to a two-way ANOVA. Again, a main effect of Knowledge was found, *F*(1, 186) = 5.40, *p* = 0.021, η^2^ = 0.028, due to participants in the informed condition giving higher ratings to the true location container (M = 35.61, SD = 27.15) than participants in the ambiguous condition (M = 25.06, SD = 18.94). There was no main effect of Protagonist, *F*(1, 186) = 0.40, *p =* 0.528, η^2^ = 0.002, or a Knowledge x Protagonist interaction, *F*(1, 186) = 0.82, *p* = 0.368, η^2^ = 0.004.

### Discussion

The results of Experiment 1 show that adults are biased by their own knowledge when reasoning about another’s false belief. Participants who were given privileged information of a hidden object’s true location (informed condition) thought it was more likely that a protagonist would look in this location, compared to participants ignorant of the true location (ambiguous condition). However, these results provide only weak support for the hypothesis that social distance modulates egocentric biases in false belief reasoning. Although numerically in line with a social distance effect, participants were not significantly less biased when reasoning about the belief of an out-group protagonist compared to an in-group protagonist. Nevertheless, the curse of knowledge effect size in the in-group condition, Cohen’s *d* = 0.468, was larger than that in the out-group condition, *d* = 0.209. The curse of knowledge effect size in the in-group condition, *d* = 0.468, is directly in line with Birch and Bloom’s [4], *d* = 0.469, and thus larger than the effect size reported by Ryskin and Brown-Schmidt [12], *d* = 0.20.

Surprisingly, participants mentalising about a heterospecific protagonist appeared to be as biased as participants mentalising about an in-group protagonist. Thus, these findings seem inconsistent with the hypothesis that increasing social distance will reduce egocentric intrusion when mentalising about a heterospecific’s false belief. However, participants in the heterospecific condition may have inferred that the dog could smell the true location of the ball. According to this notion, participants’ ratings for the true location container would be higher in the informed than in the ambiguous condition, yet the cause would not be an egocentric bias in false belief reasoning. Experiment 2 was therefore designed as a conceptual replicate of Experiment 1 that eliminated the possibility that participants inferred the dog could smell the ball’s true location. Like previously in Experiment 1, if participants were biased by their own privileged knowledge, and this curse of knowledge effect was modulated by social distance, this effect should be smaller in the out-group and, save floor effects, smaller again in the heterospecific condition, compared to the in-group condition.

## Experiment 2

### Methods

#### Participants

Participants were recruited online via Prolific (www.prolific.ac) and were of white Western European descent, aged between 18 and 35, and either UK or US nationals. Three hundred participants (50% male), who did not participate in Experiment 1, were recruited and randomly assigned to one of six conditions in a 3 (Protagonist: in-group, out-group, heterospecific) by 2 (Knowledge: informed, ambiguous) between-subjects design. Of the 300 participants, five failed to meet the inclusion criteria (aged 18–35, white Western European descent, UK or US nationals), five failed to complete the task and nine failed a suspicion check, leading to a final sample size of 281. Informed consent was gained from each participant, who received monetary compensation for completing the task. This study was approved by the Psychology Ethics Committee of the University of Cambridge.

#### Procedure

**Fig 3:**
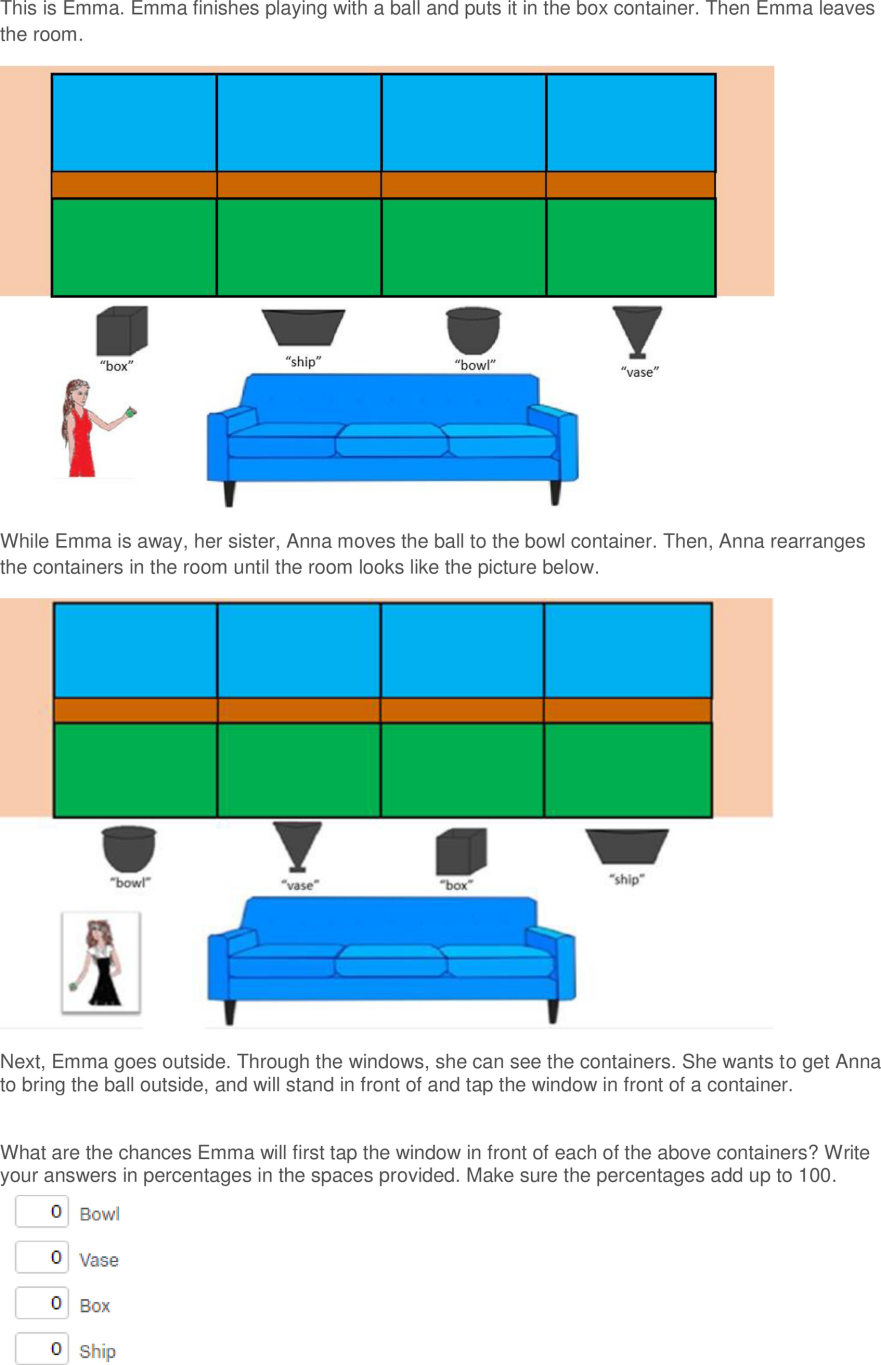
An example vignette of Experiments 2 and 3. The vignette presented to female participants in the in-group informed condition.

The procedure was identical to Experiment 1, except that the vignette was altered such that the four containers were now located between the sofa and four large windows, which looked out over a garden (see example vignette in Fig 3 and S3 and S4 Figs in the Supporting Information for examples of the other conditions). Participants read about the protagonist placing the ball in one of the containers, e.g. the “box” container. The participants were then informed that while the protagonist was not in the room, their sibling/owner moved the ball to either another, specific container, e.g. the “the bowl container” (informed condition) or another, unspecified container, i.e. “another container” (ambiguous condition), and then switched the locations of all containers. Next, participants were told the protagonist wanted the ball and would approach and tap the window in front of a container. Participants rated the percentage likelihood that the protagonist would first tap the window in front of each of the four containers. In the heterospecific condition, the dog would scratch, rather than tap, the window. This window design of Experiment 2 ensured that any apparent bias in the heterospecific condition could not be attributable to participants inferring the dog could smell the true location of the ball.

As per Experiment 1, participants typed their answers in four labelled boxes (each indicating one of the differently shaped containers) below the panel, the order of which was randomised across participants, and the programme did not allow them to continue until their answers totalled 100. The order in which the boxes and the corresponding containers were presented was randomised between participants. Within each condition, the position of the relevant containers, and the location where the ball was first placed was counterbalanced across participants, in an equivalent way to Experiment 1 (S1 Table in the Supporting Information).

#### Analysis

As per Experiment 1, we analysed the ratings for the container that the ball was moved to in the informed condition (the bowl container in Fig 3, henceforth the “true location container”). A Levene test was used to assess the homogeneity of variance of data between groups of participants, and–if appropriate–data were transformed, using the procedure outlined by Warton and Hui [23] for the logit transformation of data including 0 and 1 values. For all analyses where logit transformed data were used, the corresponding analyses using untransformed data are presented in S1 Text in the Supporting Information. For all tests that were significant, or approached significance, the transformed data analyses provided more conservative results than the raw data analyses. Participants’ ratings of the true location container were submitted to a two-way ANOVA, with Knowledge and Protagonist as between-subject factors. If, as predicted, participants were more biased by their own privileged knowledge in the in-group condition, rather than out-group or heterospecific conditions, a Knowledge x Protagonist interaction should show that the difference in participants’ ratings of the true location container between the informed and ambiguous conditions in the in-group condition was larger than in the out-group and heterospecific conditions. Post-hoc tests were conducted using independent samples t-tests with a Šidàk correction for multiple comparisons [24].

### Results

The variances of the true location container ratings were unequal across conditions (Levene test, *F*(5, 275) = 2.22, *p* = 0.052), thus data were logit transformed [23]. Transformed data were submitted to a two-way ANOVA which yielded a Knowledge x Protagonist interaction term that approached significance, *F*(2, 275) = 2.35, *p* = 0.098, η^2^ = 0.017. This was driven by participants’ ratings for the true location container being higher in the informed condition than the ambiguous condition in the in-group condition (independent samples t-test, *t*(1, 89) = 2.72, *p*_*Šidàk*_ = 0.023, *d* = 0.572), but not the out-group (independent samples t-test, *t*(1, 93) = -0.14, *p*_*Šidàk*_ = 1.00, *d* = -0.028), or heterospecific conditions (independent samples t-test, *t*(1, 93) = 0.03, *p*_*Šidàk*_ = 1.00, *d* = 0.007) (Table 2, ratings for all other containers are provided in S1 Text in the Supporting Information). No main effects of Knowledge, *F*(1, 275) = 1.90, *p* = 0.17, or Protagonist, *F*(1, 275) = 1.09, *p =* 0.34, were found.

**Table 2:** Mean (M) and standard deviation (SD) of participants’ ratings of the true location container in Experiment 2.

Knowledge	In-group	Protagonist Out-group	Heterospecific
Ambiguous	22.24 (19.65)	33.79 (29.62)	33.43 (23.94)
Informed	36.79 (25.80)*	32.60 (30.74)	34.31 (22.98)
Curse of Knowledge Cohen’s *d—*logit	0.570	-0.028	0.007
Curse of Knowledge Cohen’s *d—*raw	0.629	-0.039	0.038

The “Knowledge” rows refer to the two manipulations of the participants’ own knowledge about the object’s final location was manipulated (“Ambiguous” and “Informed”). The “Protagonist” columns refer to the three manipulations of the social distance between the participants and the protagonist (“In-group”, “Out-group”, and “Heterospecific”). The last two rows give the effect sizes for the curse of knowledge effect (the difference between participants’ ratings in the ambiguous and informed conditions for each protagonist), which were calculated both using the logit transformed data used in the statistical analyses, and from the raw data, following [4] and [12].

* denotes a mean in the informed condition that differed significantly from the ambiguous condition at α = 0.05.

### Discussion

The results from Experiment 2 provide some support for the hypothesis that social distance modulates egocentric biases in false belief reasoning. Participants only displayed an egocentric bias when mentalising about the false belief of an in-group, but not an out-group or heterospecific, protagonist. The lack of an egocentric bias in the heterospecific condition suggests that the apparent bias in Experiment 1 can be attributed to participants inferring the dog could smell the true location of the ball.

However, although there was no numerical egocentric bias in either the out-group or heterospecific conditions, the overall social distance effect, represented by the Knowledge x Protagonist interaction term, was not significant. Hence, to follow up on the directional results observed in Experiments 1 and 2, we conducted a replication of Experiment 2 with a sample size designed to achieve 90% power to detect an effect size of η^2^ = 0.017, which was the effect size observed in Experiment 2.

## Experiment 3

### Methods

#### Participants

Participants were recruited online via Prolific (www.prolific.ac) and were of white Western European descent, aged between 18 and 35, and either UK or US nationals. Seven hundred and eighty participants (50% male), who had not participated in Experiments 1 or 2, were recruited and randomly assigned to one of six conditions in a 3 (Protagonist: in-group, out-group, heterospecific) by 2 (Knowledge: informed, ambiguous) between-subjects design. Of the 780 participants, 25 failed to meet the inclusion criteria (aged 18–35, white Western European descent, UK or US nationals), two failed to complete the task and nine failed a suspicion check, leading to a final sample size of 744. This sample size has 90.4% power to detect an effect of η^2^ = 0.017 in our 3 x 2 between-subjects design. Informed consent was gained from each participant, who received monetary compensation for completing the task. This study was approved by the Psychology Ethics Committee of the University of Cambridge.

#### Procedure and analyses

The procedure and analyses were identical to Experiment 2.

### Results

The variances of the true location container ratings were unequal across conditions (Levene test, *F*(5, 738) = 10.40, *p* < 0.001), thus data were logit transformed [23]. The variances of the transformed data were still unequal, but to a lesser extent than the raw data (Levene test, *F*(5, 738) = 3.46, *p* = 0.004). Logit transformed data were submitted to a two-way ANOVA. A main effect of Knowledge was found, *F*(1, 738) = 22.30, *p* < 0.001, η^2^ = 0.029, but there was no significant effect of Protagonist, *F*(2, 738) = 0.97, *p* = 0.38, η^2^ = 0.003, or a significant Knowledge x Protagonist interaction, *F*(2, 738) = 0.01, *p* = 0.99, η^2^ < 0.001. The main effect of Knowledge was driven by participants in the informed condition giving higher ratings to the true location container (M = 40.10, SD = 29.55) than participants in the ambiguous condition (M = 29.45, SD = 23.15) (Table 3, ratings for all other containers are provided in S1 Text in the Supporting Information).

**Table 3:** Mean (M) and standard deviation (SD) of participants’ ratings of the true location container in Experiment 3.

Knowledge	In-group	Protagonist Out-group	Heterospecific
Ambiguous	28.37 (22.93)	27.73 (21.29)	32.28 (25.02)
Informed***	41,03 (32.14)	37.66 (29.69)	41.61 (26.70)
Curse of Knowledge Cohen’s *d—*logit	0.332	0.323	0.384
Curse of Knowledge Cohen’s *d—*raw	0.455	0.385	0.361

The “Knowledge” rows refer to the two manipulations of the participants’ own knowledge about the object’s final location was manipulated (“Ambiguous” and “Informed”). The “Protagonist” columns refer to the three manipulations of the social distance between the participants and the protagonist (“In-group”, “Out-group”, and “Heterospecific”). The last two rows give the effect sizes for the curse of knowledge effect (the difference between participants’ ratings in the ambiguous and informed conditions for each protagonist), which were calculated both using the logit transformed data used in the statistical analyses, and from the raw data, following [4] and [12].

*** denotes that the mean across the informed conditions differed significantly from the ambiguous conditions at α = 0.001.

### Discussion

The results from Experiment 3, a direct replication of Experiment 2 with a larger sample size, are not in-line with the hypothesis that social distance modulates egocentric biases in false belief reasoning. Participants displayed an egocentric bias of similar magnitude when mentalising about the false belief of in-group, out-group and heterospecific protagonists. The curse of knowledge effect size from the logit data, Cohen’s *d* = 0.332, in the in-group condition, was smaller than that reported by Birch and Bloom, *d* = 0.469 [4]. However, when calculating the effect size from the raw data, as per Birch and Bloom, the curse of knowledge effect, *d* = 0.455, was similar to the original study.

To summarise the findings of our three experiments we then conducted, i) an internal meta-analytic summary of the results of these three experiments and ii) a meta-analysis of these three experiments and Todd et al. [11], to provide a best estimate for the magnitude of the curse of knowledge and social distance effects when adults reason about the false beliefs of human in-group and out-group protagonists [25,26]. Multivariate random-effects meta-analyses were performed using the package ‘metafor’ in R 3.4.2 [27], with Knowledge and Protagonist as moderators, and Experiment as a random factor. The meta-analyses were performed on logit transformed data, and the complementary analyses on raw data are presented in S1 Text. Whenever the raw analysis differed from the logit-transformed analysis is highlighted in the main text.

## Meta-analyses

### Results

The meta-analytic summary of Experiments 1, 2 and 3 revealed a significant overall Knowledge effect, β (raw effect estimate, logit transformed data) = 0.36; 95% CI = [0.19, 0.53], z = 4.20, *p* < 0.0001. There was no significant effect of Protagonist, β = 0.038; 95% CI = [-0.11, 0.19], z = 0.51, *p* = 0.61, and no significant interaction, β = -0.14; 95% CI = [-0.38, 0.09], z = -1.19, *p* = 0.23. There was no evidence of any significant effects of residual heterogeneity, Q(8) = 7.68, p = 0.47. Overall, the best estimate of the curse of knowledge effect across all conditions was, Cohen’s *d* = 0.314. Individually, the best estimates of the curse of knowledge effect in the in-group, Cohen’s *d* = 0.407 and out-group, Cohen’s *d* = 0.224, conditions. The pattern of significance was the same in the raw data analysis, however effect sizes were overall larger in the raw data analysis: overall *d* = 0.394, in-group *d* = 0.507, out-group *d* = 0.285.

When the data from Todd et al. ([11]: Experiment 4) were included, there was again a significant overall Knowledge effect, β = 0.38; 95% CI = [0.22, 0.54], z = 4.70, *p* < 0.0001, and no significant effect of Protagonist, M = 0.1580; 95% CI = [-0.0373, 0.3532], z = 1.5854, *p* = 0.1129. By contrast to our internal meta-analysis, here the interaction term was significant, β = -0.23; 95% CI = [-0.45, -0.0087], z = -2.04, *p* = 0.042. This was driven by a significant curse of knowledge effect across the four experiments in the in-group condition, Cohen’s *d* = 0.428, 95% CI (0.23, 0.63), z = 4.17, *p <* 0.001, but not the out-group condition, Cohen’s *d* = 0.145, 95% CI (-0.056, 0.34), z = 1.42, *p* = 0.16. By contrast, in the raw data analysis, the curse of knowledge effect approached significance across the out-group conditions, Cohen’s *d* = 0.195, 95% CI (-0.014, 0.40), z = 1.83, *p* = 0.068. There was evidence of residual heterogeneity, Q(12) = 44.58, *p* < 0.001, which justified the selection of a random effects meta-analysis, as study specific variables between our own and Todd et al.’s studies could have influenced the curse of knowledge or social distance effects [28].

### Discussion

The internal meta-analysis of the current study does not support the hypothesis that social distance modulates egocentric biases in false belief reasoning. While the data are numerically in-line with the prediction that participants would display smaller egocentric biases when mentalising about out-group, rather than in-group, members, this effect was not significant across a large sample size (N = 872 across the conspecific conditions). To enable comparisons with previous studies, the best estimate of the curse of knowledge effect size across all conspecific conditions of the current study, Cohen’s *d* = 0.314, was smaller than originally reported by Birch and Bloom [4], *d* = 0.469, and larger than reported by Ryskin and Brown-Schmidt [12], *d* = 0.20. However, when the effect size was calculated from our raw data, following the methods of Birch and Bloom, and Ryskin and Brown-Schmidt, the curse of knowledge effect size was *d* = 0.507 in the in-group condition, directly in-line with Birch and Bloom.

By contrast, when the data from Todd et al. [11] were included, the social distance effect was significant. This may suggest that an effect of social distance may be dependent on the specific relationship between perspective takers and their targets. Consistent with this idea, Mullen et al. reported that using more salient out-group manipulations reduced the amount of social projection of US students onto these targets ([29], see also [30]). Hence, if the Turkish-German out-group manipulation used by Todd et al. [11] was more salient than our Western African–white UK/US out-group manipulation, this could explain the apparent discrepancies in our results. There are several lines of evidence to suggest the Turkish-German out-group may be particularly salient. For example, there is a relatively large Turkish population in Germany and there are many documented cases of differences in behaviour of Germans to Turkish out-group members (e.g. [31,32]). Even categorising robots as Turkish, compared with German, has been shown to reduce favourability ratings and anthropomorphism in German students [33]. Therefore, the difference in our own and Todd et al.’s results could be due to the different *type* of out-group manipulation being used.

## General discussion

This study tested i) whether adults were biased by their own privileged knowledge when reasoning about another’s false belief, ii) whether the magnitude of this egocentric bias was modulated by social distance and, iii) whether such a social distance effect was still observed with a heterospecific out-group. Across three experiments, participants were biased by their own privileged knowledge when reasoning about the false beliefs of in-group, out-group and heterospecific protagonists. Whilst the data were numerically in-line with the hypothesis that increasing social distance reduces egocentric biases when reasoning about false beliefs, the effect was not significant in i) any of the three studies individually, or ii) overall upon internal meta-analysis. Therefore, the present data do not support the hypothesis that social distance modulates adults’ egocentric biases when reasoning about false beliefs.

Our results suggest the social distance effect is unlikely to explain the smaller curse of knowledge effect size observed by Ryskin and Brown-Schmidt [12], Cohen’s *d* = 0.20, than that originally reported by Birch and Bloom [4], *d* = 0.469. Nevertheless, the curse of knowledge effect size across both conspecific conditions in the current study, *d* = 0.314, is larger than Ryskin and Brown-Schmidt’s, and, across the in-group conditions, the curse of knowledge effect was replicated with effect sizes directly in line with Birch and Bloom [4], namely *d* = 0.407 from the logit transformed analysis, and *d* = 0.507 from the raw data analysis. Therefore, the modified false belief task does seem to reliably elicit egocentric biases in false belief reasoning when participants of white Western European descent mentalise about an in-group protagonist.

Further, our finding of a significant curse of knowledge effect across the out-group conditions of the current study suggest egocentric intrusion might not be limited to mentalising about in-group members. This diverges from some of the previous literature, in which either no, or negative, egocentric biases were observed when adults reasoned about the preferences [6], visceral states [16], knowledge [11] or traits [13] of out-group members and dissimilar others. In particular, that our results contrasted with Todd et al.’s [11], who used a highly similar paradigm to us, suggests that the specifics of an in-group/out-group relationship may influence whether participants are egocentrically biased when mentalising about an out-group member.

That participants do sometimes display egocentric biases when mentalising about out-group members is not unexpected. For example, a small degree of egocentric intrusion when reasoning about out-group members is often reported in studies examining social projection [30]. However, in contrast to our findings, the degree of social projection is significantly smaller towards out-group members, compared with in-group members, across these studies [30]. This reinforces the possibility that the degree of egocentric intrusion experienced when taking the perspective of out-group members is both task and situation dependent. For example, it is possible that different mechanisms are used across different facets of Theory of Mind, and these may vary with social distance. In-line with this suggestion, Ames [34] showed that participants engaged in more stereotyping, and less self-projection, when judging the attitudes and desires of a protagonist perceived to be dissimilar to them.

That the salience of an out-group could modulate the effects of egocentric intrusion in Theory of Mind highlights the importance of using multiple out-groups before results can be interpreted as a *general* feature of intergroup cognition. Further, with accumulating evidence that socio-cultural factors may influence perspective taking [11,13,17,35–37], results from one participant base may not generalise to another. Such cultural differences in participant bases might explain some of the difference in the curse of knowledge effect size between our data and Ryskin and Brown-Schmidt’s [12]. As well as being ethnically heterogenous, their sample was likely more culturally heterogeneous than our own. Conversely, it is perhaps unlikely that a single factor such as culture is sufficient to explain all of the difference between our results and Ryskin and Brown-Schmidt’s, as the two studies differed in more than just the culture and ethnicity of the participants. For example, participants’ experience with psychological tests, or proficiency in English, may have differed between our white UK/US sample and Ryskin and Brown-Schmidt’s sample of either undergraduates in the United States, or Amazon Mechanical Turk workers from the United States, and these in turn could have influenced the magnitude of any egocentric bias.

Also warranting further investigation is which factors contribute to making an out-group ‘salient’, particularly with regards to modulating egocentric intrusion in Theory of Mind. A host of correlated features, such as likeability, similarity, familiarity and group-membership have been proposed as potential modulators of egocentric intrusion (see [13] for discussion). These features are difficult to disentangle using human out-groups, although recent studies suggest inter-personal dissimilarity may account for reductions in social projection [13,17]. However, the presence of an egocentric bias in the heterospecific condition in the current study suggests that inter-agent dissimilarity is not on its own sufficient to reduce egocentric biases when reasoning about false beliefs. Furthermore, such heterospecific protagonists may offer a unique method to test and validate theories of perspective taking. As examples, heterospecific protagonists may, i) facilitate the dissociation of the impacts of likeability, similarity, familiarity and group-membership on egocentric biases in Theory of Mind, ii) offer a window to minimise the effect of socio-cultural factors on participants while still using an animate protagonist, and iii) provide an interesting contrast between inanimate non-anthropomorphised stimuli, such as arrows (e.g. [38]), inanimate anthropomorphised stimuli, such as robots (e.g. [33]), and animate human protagonists.

## Conclusions

Egocentric biases in false belief reasoning were reliably observed across three conceptual replicates of the Birch and Bloom [4] modified false belief task, when US and UK adults mentalised about in-group, out-group and heterospecific protagonists. These findings are not in-line with the hypothesis that the magnitude of such biases is modulated by social distance [10,11], and suggest inter-agent dissimilarity alone is not sufficient to modulate egocentric biases when reasoning about false beliefs.

## Supporting information

S1 Fig An example vignette of the out-group ambiguous condition of Experiment 1. Links to the Qualtrics project containing all vignettes available from the author.
(TIF)

S2 Fig An example vignette of the heterospecific informed condition of Experiment 1. (TIF)

S3 Fig An example vignette of the out-group ambiguous condition of Experiments 2 and 3. (TIF)

S4 Fig An example vignette of the heterospecific informed condition of Experiments 2 and 3. (TIF)

S1 Table Counterbalancing of containers between participants in Experiments 1, 2 and 3. (DOCX)

S1 Text Participants’ ratings of each container in Experiments 1, 2 and 3, and the results of the raw data analysis from Experiments 1, 2 and 3, and the meta-analyses. Raw data, and analysis scripts, are provided at: https://osf.io/xm94v/.
(DOCX)

## References

[1] Epley N, Morewedge CK, Keysar B. Perspective taking in children and adults: Equivalent egocentrism but differential correction. Journal of Experimental Social Psychology. 2004 11 1;40(6):760–8.

[2] Van Boven L, Loewenstein G. Social projection of transient drive states. Personality and Social Psychology Bulletin. 2003 9;29(9):1159–68. doi: 10.1177/0146167203254597 15189611 10.1177/0146167203254597

[3] Camerer C, Loewenstein G, Weber M. The curse of knowledge in economic settings: An experimental analysis. Journal of Political Economy. 1989 10 1;97(5):1232–54.

[4] Birch SA, Bloom P. The curse of knowledge in reasoning about false beliefs. Psychological Science. 2007 5;18(5):382–6. doi: 10.1111/j.1467-9280.2007.01909.x 17576275 10.1111/j.1467-9280.2007.01909.x

[5] Epley N, Keysar B, Van Boven L, Gilovich T. Perspective taking as egocentric anchoring and adjustment. Journal of Personality and Social Psychology. 2004 9;87(3):327 doi: 10.1037/0022-3514.87.3.327 15382983 10.1037/0022-3514.87.3.327

[6] Tamir DI, Mitchell JP. Anchoring and adjustment during social inferences. Journal of Experimental Psychology: General. 2013 2;142(1):151. 22506753 10.1037/a0028232

[7] Sommerville JA, Bernstein DM, Meltzoff AN. Measuring beliefs in centimeters: Private knowledge biases preschoolers' and adults' representation of others' beliefs. Child Development. 2013 11 1;84(6):1846–54. doi: 10.1111/cdev.12110 23581849 10.1111/cdev.12110

[8] Converse BA, Lin S, Keysar B, Epley N. In the mood to get over yourself: mood affects theory-of-mind use. Emotion. 2008 10;8(5):725 doi: 10.1037/a0013283 18837624 10.1037/a0013283

[9] Dębska A, Komorowska K. Limitations in reasoning about false beliefs in adults: the effect of priming or the curse of knowledge?. Psychology of Language and Communication. 2013 12 1;17(3):269–78.

[10] Sassenrath C, Sassenberg K, Semin GR. Cool, but understanding… Experiencing cooler temperatures promotes perspective-taking performance. Acta Psychologica. 2013 6 30;143(2):245–51. doi: 10.1016/j.actpsy.2013.03.011 23644580 10.1016/j.actpsy.2013.03.011

[11] Todd AR, Hanko K, Galinsky AD, Mussweiler T. When focusing on differences leads to similar perspectives. Psychological Science. 2011 1;22(1):134–41. doi: 10.1177/0956797610392929 21156862 10.1177/0956797610392929

[12] Ryskin RA, Brown-Schmidt S. Do adults show a curse of knowledge in false-belief reasoning? A robust estimate of the true effect size. PloS ONE. 2014 3 25;9(3):e92406 doi: 10.1371/journal.pone.0092406 24667826 10.1371/journal.pone.0092406 PMC3965426

[13] Davis MH. Social projection to liked and disliked targets: The role of perceived similarity. Journal of Experimental Social Psychology. 2017 5 31;70:286–93.

[14] Epley N, Converse BA, Delbosc A, Monteleone GA, Cacioppo JT. Believers' estimates of God's beliefs are more egocentric than estimates of other people's beliefs. Proceedings of the National Academy of Sciences. 2009 12 22;106(51):21533–8. 10.1073/pnas.0908374106 PMC2787468 19955414

[15] Lee WS, Atance CM. The Effect of Psychological Distance on Children’s Reasoning about Future Preferences. PloS ONE. 2016 10 14;11(10):e0164382 doi: 10.1371/journal.pone.0164382 27741264 10.1371/journal.pone.0164382 PMC5065213

[16] O’Brien E, Ellsworth PC. More than skin deep: Visceral states are not projected onto dissimilar others. Psychological Science. 2012 4;23(4):391–6. doi: 10.1177/0956797611432179 22402799 10.1177/0956797611432179

[17] Simpson AJ, Todd AR. Intergroup visual perspective-taking: Shared group membership impairs self-perspective inhibition but may facilitate perspective calculation. Cognition. 2017 9 30;166:371–81. doi: 10.1016/j.cognition.2017.06.003 28605699 10.1016/j.cognition.2017.06.003

[18] Zhong CB, Leonardelli GJ. Cold and lonely: Does social exclusion literally feel cold?. Psychological Science. 2008 9;19(9):838–42. doi: 10.1111/j.1467-9280.2008.02165.x 18947346 10.1111/j.1467-9280.2008.02165.x

[19] Francis G. The frequency of excess success for articles in Psychological Science. Psychonomic Bulletin & Review. 2014 10 1;21(5):1180–7. 24638826 10.3758/s13423-014-0601-x

[20] Pettigrew TF. Intergroup contact theory. Annual Review of Psychology. 1998 2;49(1):65–85. 10.1146/annurev.psych.49.1.65 15012467

[21] Zajonc RB. Attitudinal effects of mere exposure. Journal of Personality and Social Psychology. 1968 6;9(2p2):1.5667435

[22] Eddy TJ, Gallup GG, Povinelli DJ. Attribution of cognitive states to animals: Anthropomorphism in comparative perspective. Journal of Social Issues. 1993 4 1;49(1):87–101.

[23] Warton DI, Hui FK. The arcsine is asinine: the analysis of proportions in ecology. Ecology. 2011 1 1;92(1):3–10. 21560670 10.1890/10-0340.1

[24] Šidák Z. Rectangular confidence regions for the means of multivariate normal distributions. Journal of the American Statistical Association. 1967 6 1;62(318):626–33.

[25] Cumming G. The new statistics: Why and how. Psychological science. 2014 1;25(1):7–29. doi: 10.1177/0956797613504966 24220629 10.1177/0956797613504966

[26] McShane BB, Böckenholt U. Single-Paper Meta-Analysis: Benefits for Study Summary, Theory Testing, and Replicability. Journal of Consumer Research. 2017 4 1;43(6):1048–63.

[27] Viechtbauer W. Conducting meta-analyses in R with the metafor package. J Stat Softw. 2010 8 5;36(3):1–48.

[28] Ueno T, Fastrich GM, Murayama K. Meta-analysis to integrate effect sizes within an article: Possible misuse and Type I error inflation. Journal of Experimental Psychology: General. 2016 5;145(5):643. 27019021 10.1037/xge0000159

[29] Mullen B, Dovidio JF, Johnson C, Copper C. In-group-out-group differences in social projection. Journal of Experimental Social Psychology. 1992 9 1;28(5):422–40.

[30] Robbins JM, Krueger JI. Social projection to ingroups and outgroups: A review and meta-analysis. Personality and Social Psychology Review. 2005 2;9(1):32–47. doi: 10.1207/s15327957pspr0901_3 15745863 10.1207/s15327957pspr0901_3

[31] Florack A, Scarabis M, Bless H. When do associations matter? The use of automatic associations toward ethnic groups in person judgments. Journal of Experimental Social Psychology. 2001 11 1;37(6):518–24.

[32] Kaas L, Manger C. Ethnic discrimination in Germany's labour market: a field experiment. German Economic Review. 2012 2 1;13(1):1–20.

[33] Eyssel F, Kuchenbrandt D. Social categorization of social robots: Anthropomorphism as a function of robot group membership. British Journal of Social Psychology. 2012 12 1;51(4):724–31. doi: 10.1111/j.2044-8309.2011.02082.x 22103234 10.1111/j.2044-8309.2011.02082.x

[34] Ames DR. Inside the mind reader's tool kit: projection and stereotyping in mental state inference. Journal of Personality and Social Psychology. 2004 9;87(3):340 doi: 10.1037/0022-3514.87.3.340 15382984 10.1037/0022-3514.87.3.340

[35] Kessler K, Cao L, O'Shea KJ, Wang H. A cross-culture, cross-gender comparison of perspective taking mechanisms. Proceedings of the Royal Society of London B: Biological Sciences. 2014 6 22;281(1785):20140388. 10.1098/rspb.2014.0388 PMC4024296 24807256

[36] Wu S, Keysar B. The effect of culture on perspective taking. Psychological Science. 2007 7;18(7):600–6. doi: 10.1111/j.1467-9280.2007.01946.x 17614868 10.1111/j.1467-9280.2007.01946.x

[37] Wu S, Barr DJ, Gann TM, Keysar B. How culture influences perspective taking: differences in correction, not integration. Frontiers in Human Neuroscience. 2013;7. 10.3389/fnhum.2013.00822 PMC3845341 24348368

[38] Santiesteban I, Catmur C, Hopkins SC, Bird G, Heyes C. Avatars and arrows: Implicit mentalizing or domain-general processing?. Journal of Experimental Psychology: Human Perception and Performance. 2014 6;40(3):929 doi: 10.1037/a0035175 24377486 10.1037/a0035175

