# Supplementary figures and images for: Does social distance modulate adults’ egocentric biases when reasoning about false beliefs?
Source: PLoS One. 2018 Jun 8;13(6):e0198616. doi: 10.1371/journal.pone.0198616 (PMC5993257; doi:10.1371/journal.pone.0198616)

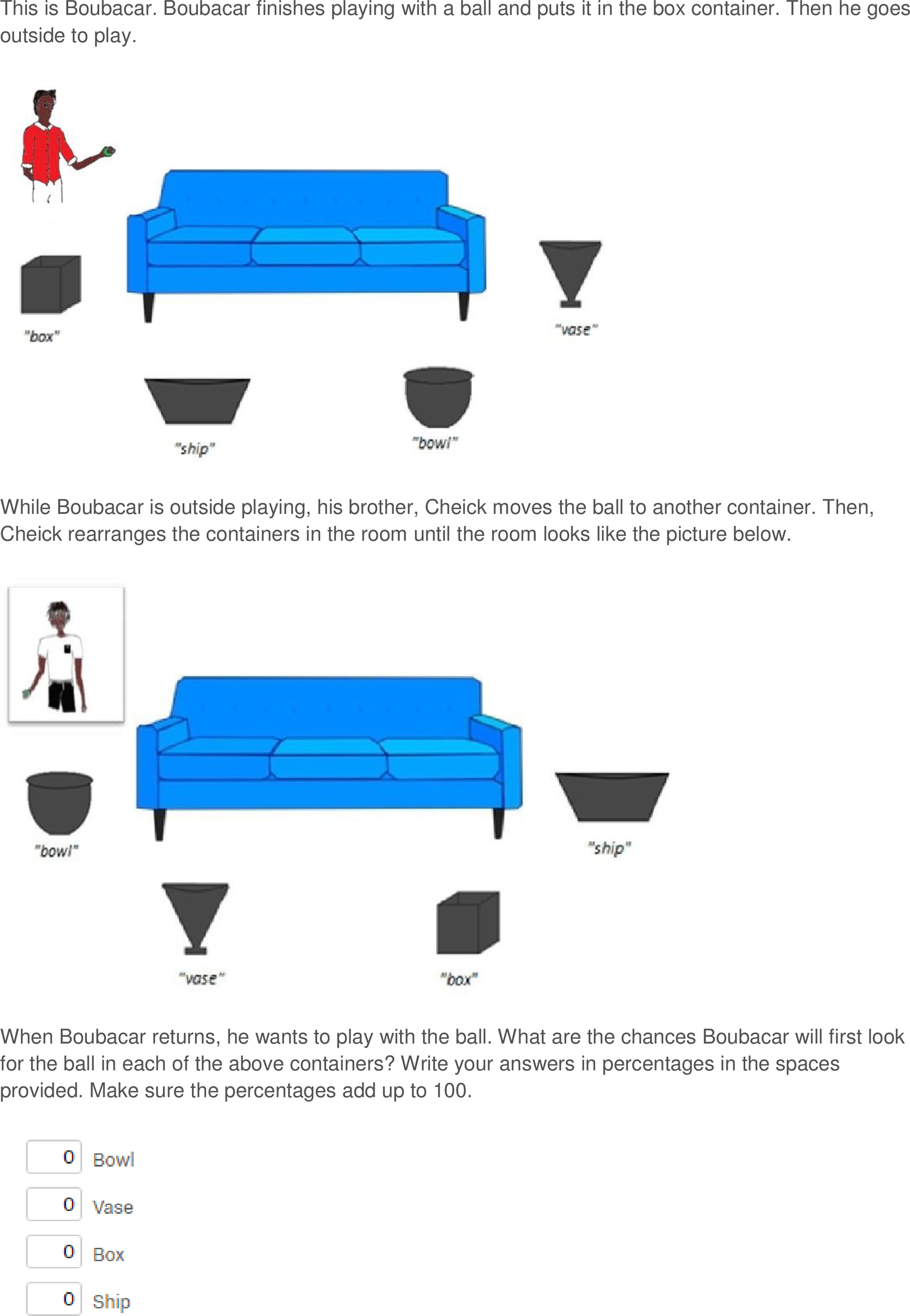

Supplement: S1 Fig — Links to the Qualtrics project containing all vignettes available from the author. (TIF) [file pone.0198616.s001.tif]

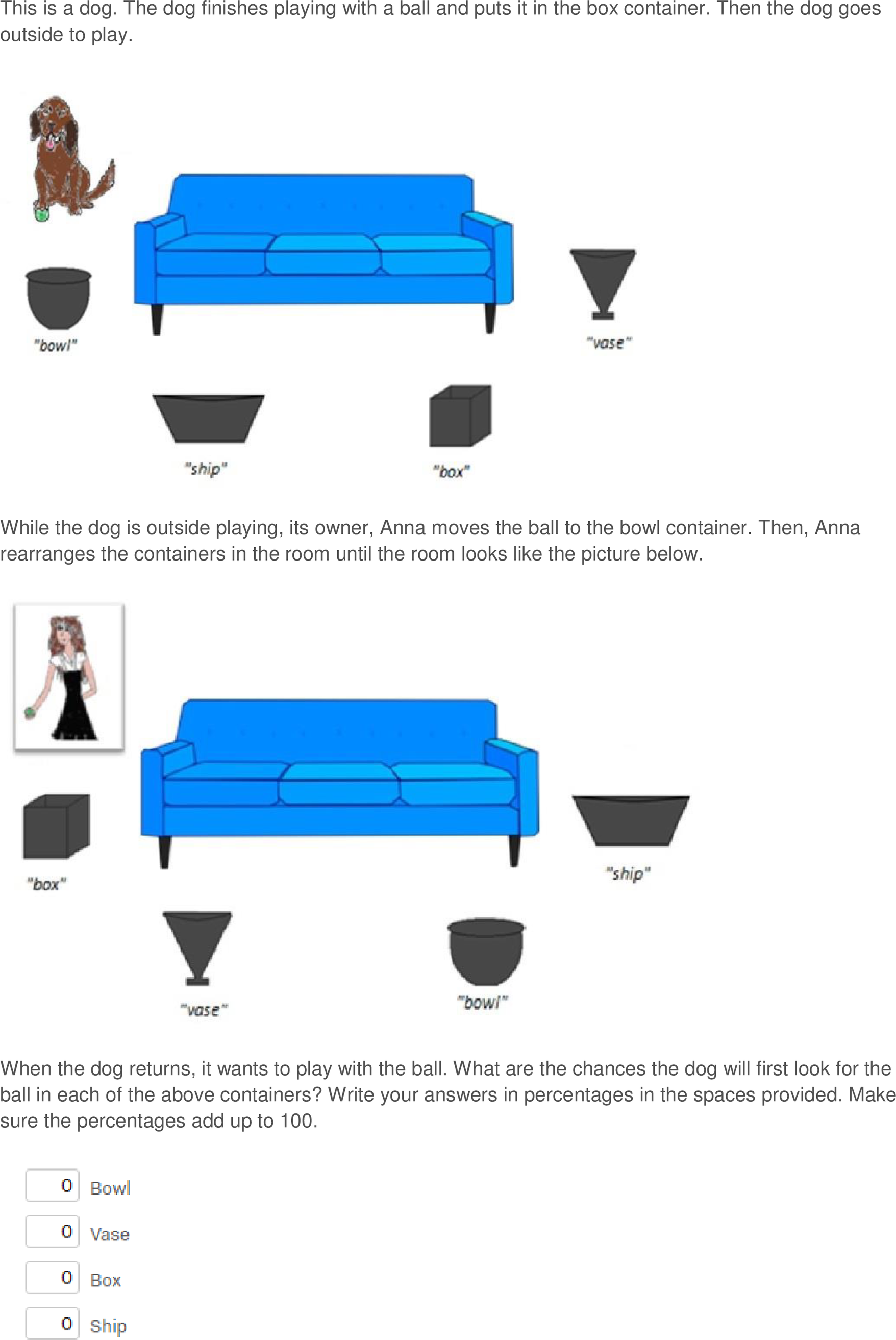

Supplement: S2 Fig — (TIF) [file pone.0198616.s002.tif]

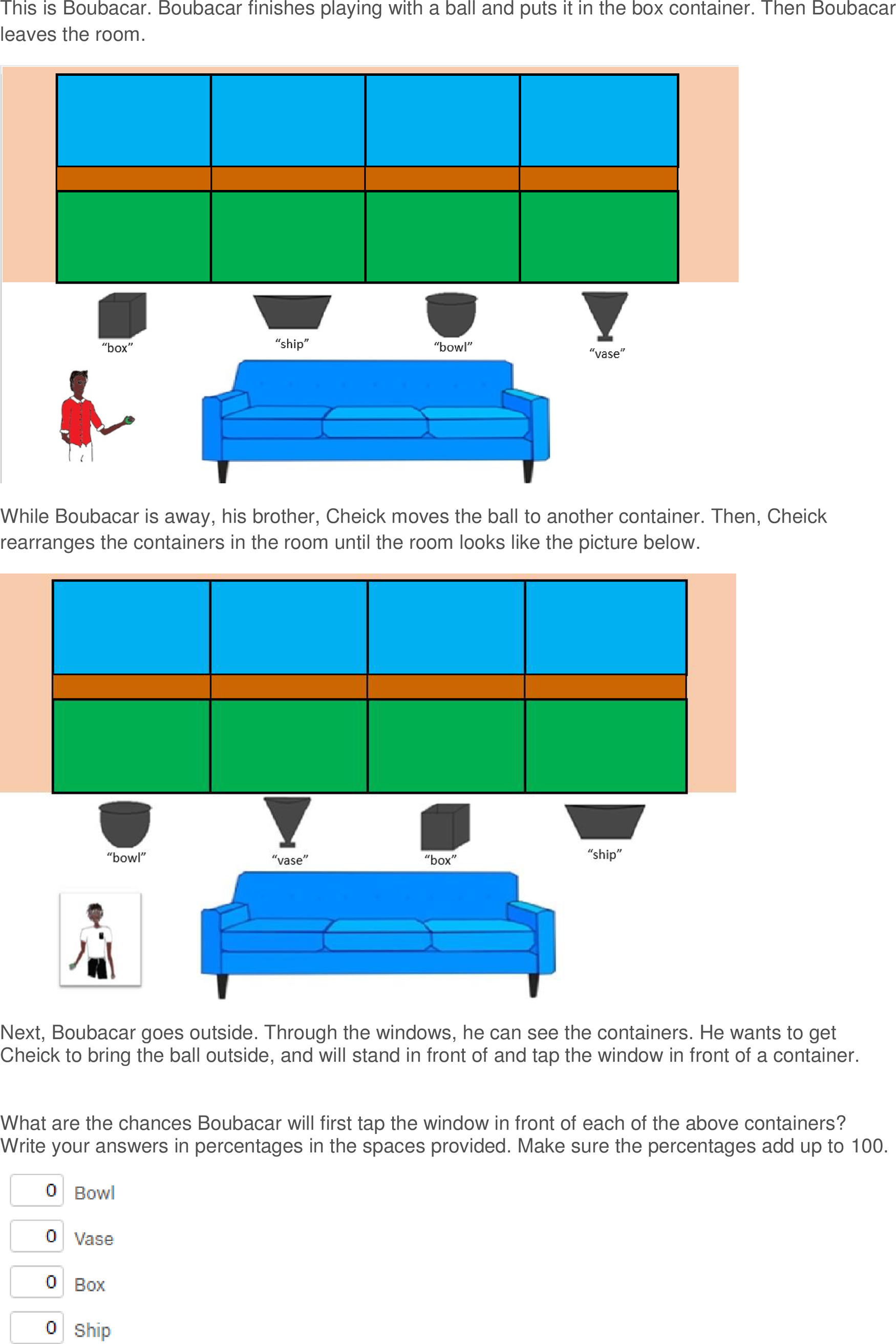

Supplement: S3 Fig — (TIF) [file pone.0198616.s003.tif]

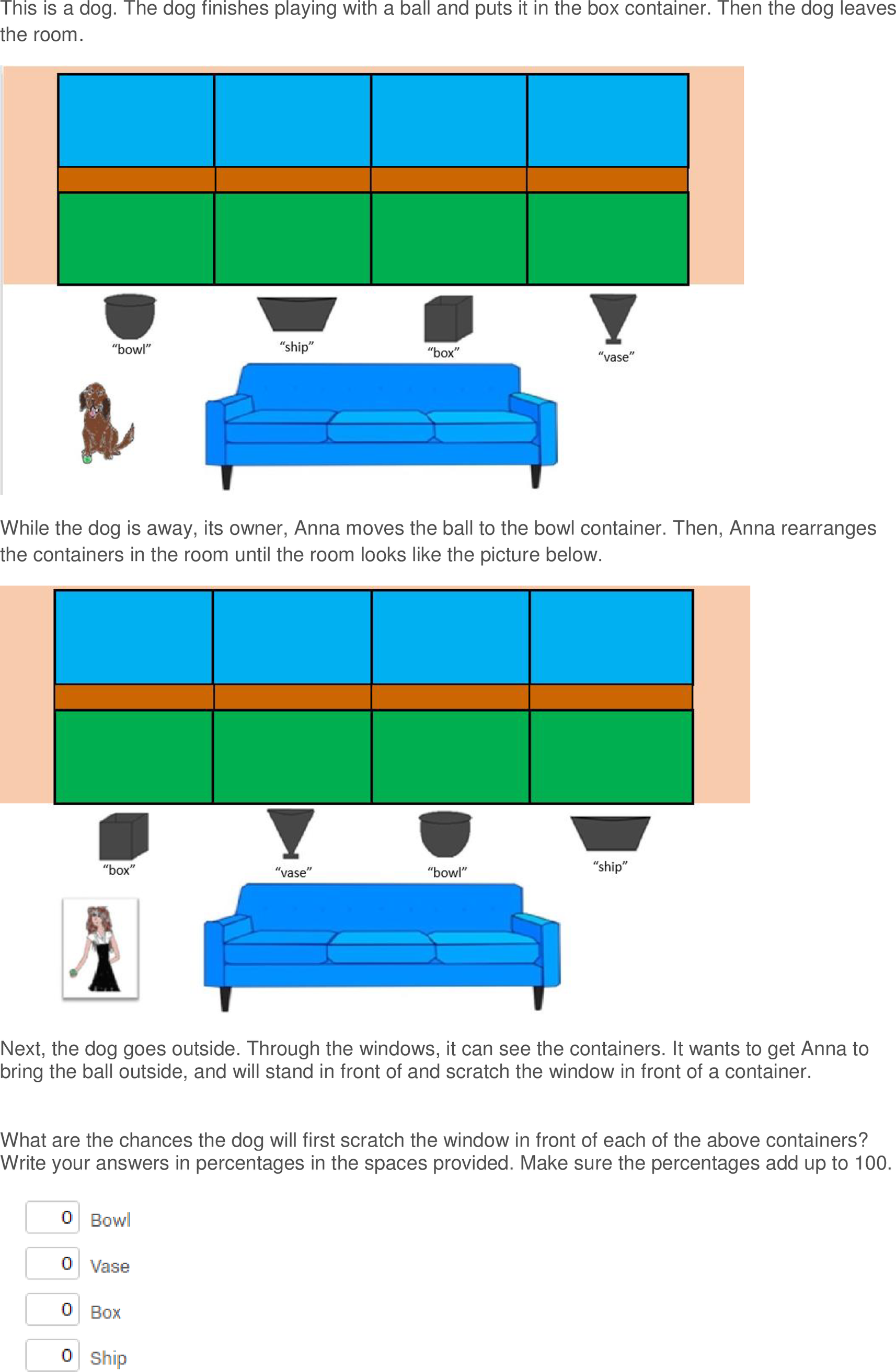

Supplement: S4 Fig — (TIF) [file pone.0198616.s004.tif]
