# Supplementary material for: Does social distance modulate adults’ egocentric biases when reasoning about false beliefs?
Source: PLoS One. 2018 Jun 8;13(6):e0198616. doi: 10.1371/journal.pone.0198616 (PMC5993257; doi:10.1371/journal.pone.0198616)
Supplement: S1 Table — (DOCX) [file pone.0198616.s005.docx]

**S1 Table: Counterbalancing of containers between participants in Experiments 1, 2 and 3.**

| Proportion of trials | 25% | 25% | 25% | 25% |
| --- | --- | --- | --- | --- |
| Order Panel 1 | **Box**, Ship, Bowl, Vase | Bx, S, **Bw**, V | **Bw**, S, Bx, V | Bw, S, **Bx**, V |
| Order Panel 2 | **Bowl**, Vase, Box, Ship | Bw, V, **Bx**, S | **Bx**, V, Bw, S | Bx, V, **Bw**, S |

The location of the ball was first placed is denoted by the container being in bold type. Bx = Box, S = Ship, Bw = Bowl, V = Vase.
