# Supplementary material for: Does social distance modulate adults’ egocentric biases when reasoning about false beliefs?
Source: PLoS One. 2018 Jun 8;13(6):e0198616. doi: 10.1371/journal.pone.0198616 (PMC5993257; doi:10.1371/journal.pone.0198616)
Supplement: S1 Text — Raw data, and analysis scripts, are provided at: https://osf.io/xm94v/. (DOCX) [file pone.0198616.s006.docx]

**S1 Text:**

**Participants’ mean ratings of each container in Experiment 1**

False belief container (the container into which the protagonist first placed the ball): M (SD)

| **Knowledge** | In-group | **Protagonist**  Out-group | Heterospecific |
| --- | --- | --- | --- |
| Ambiguous | 55.07 (29.39) | 51.91 (29.17) | 48.93 (22.45) |
| Informed | 45.83 (30.79) | 54.69 (31.61) | 37.19 (23.02) |

Vase container:

| **Knowledge** | In-group | **Protagonist**  Out-group | Heterospecific |
| --- | --- | --- | --- |
| Ambiguous | 9.09 (10.57) | 12.34 (14.15) | 10.33 (9.64) |
| Informed | 7.67 (10.13) | 6.96 (10.47) | 10.08 (10.64) |

Ship container:

| **Knowledge** | In-group | **Protagonist**  Out-group | Heterospecific |
| --- | --- | --- | --- |
| Ambiguous | 9.96 (11.52) | 11.49 (11.43) | 9.79 (9.04) |
| Informed | 7.63 (10.65) | 5.94 (9.40) | 7.97 (9.36) |

N = Ambiguous In-group: 46 Out-group: 47 Heterospecific: 43

Informed In-group: 48 Out-group: 49 Heterospecific: 50

**Participants’ mean ratings of each container in Experiment 2**

False belief container:

| **Knowledge** | In-group | **Protagonist**  Out-group | Heterospecific |
| --- | --- | --- | --- |
| Ambiguous | 52.92 (35.58) | 44.94 (35.69) | 41.06 (25.19) |
| Informed | 47.50 (29.39) | 52.29 (35.01) | 49.40 (27.25) |

Vase container:

| **Knowledge** | In-group | **Protagonist**  Out-group | Heterospecific |
| --- | --- | --- | --- |
| Ambiguous | 11.77 (12.95) | 9.43 (12.17) | 13.11 (12.60) |
| Informed | 7.93 (10.84) | 7.19 (10.41) | 8.54 (11.05) |

Ship container:

| **Knowledge** | In-group | **Protagonist**  Out-group | Heterospecific |
| --- | --- | --- | --- |
| Ambiguous | 13.07 (13.96) | 11.85 (18.06) | 12.40 (12.03) |
| Informed | 7.79 (10.86) | 8.02 (12.11) | 7.75 (9.71) |

N = Ambiguous In-group: 49 Out-group: 47 Heterospecific: 47

Informed In-group: 42 Out-group: 48 Heterospecific: 48

**Participants’ mean ratings of each container in Experiment 3**

False belief container:

| **Knowledge** | In-group | **Protagonist**  Out-group | Heterospecific |
| --- | --- | --- | --- |
| Ambiguous | 44.68 (32.37) | 44.44 (30.16) | 48.77 (28.52) |
| Informed | 40.84 (33.38) | 41.24 (30.48) | 40.58 (26.79) |

Vase container:

| **Knowledge** | In-group | **Protagonist**  Out-group | Heterospecific |
| --- | --- | --- | --- |
| Ambiguous | 13.36 (12.47) | 13.98 (13.30) | 9.90 (12.50) |
| Informed | 9.06 (12.17) | 10.57 (11.96) | 8.74 (10.72) |

Ship container:

| **Knowledge** | In-group | **Protagonist**  Out-group | Heterospecific |
| --- | --- | --- | --- |
| Ambiguous | 13.59 (12.45) | 13.84 (12.54) | 9.06 (11.66) |
| Informed | 9.10 (13.13) | 10.53 (13.99) | 9.06 (12.17) |

N = Ambiguous In-group: 126 Out-group: 125 Heterospecific: 124

Informed In-group: 122 Out-group: 123 Heterospecific: 124

**Raw data analysis**

**Experiment 1**

There were main effects of Knowledge, *F*(1, 277) = 17.52, *p*<0.001, η^2^ = 0.058, and Protagonist, *F*(2, 277) = 4.35, *p=*0.014, η^2^ = 0.028, but no Knowledge x Protagonist interaction, (*F*(2, 277) = 0.407, *p*=0.666, η^2^ = 0.0027.

The main effect of Protagonist was driven by overall ratings being greater in the heterospecific than out-group condition (independent samples t-test, *p_Šidàk_*=0.014).

Experiment 1 – conspecific only

Again, a main effect of Knowledge was found, *F*(1, 186) = 9.56, *p*=0.0023, η^2^ = 0.0048. There was no main effect of Protagonist, *F*(1, 186) = 1.45, *p=*0.230, η^2^ = 0.0073, or a Knowledge x Protagonist interaction, *F*(1, 186) = 0.50, *p*=0.48, η^2^ = 0.0025.

**Experiment 2**

The Knowledge x Protagonist interaction term approached significance, *F*(2, 275) = 2.54, *p*=0.081, η^2^ = 0.018. There were no main effects of Knowledge, *F*(1, 275) = 2.24, *p*=0.14, η^2^ = 0.0081, or Protagonist, *F*(2, 275) = 0.99, *p=*0.37, η^2^ = 0.0066, were found.

The interaction effect was driven by ratings being higher in the informed than ambiguous condition in the in-group (independent samples t-test, *p_Šidàk_*=0.011) but not out-group (independent samples t-test, *p_Šidàk_*=1) conditions.

**Experiment 3**

There was a main effect of Knowledge, *F*(1, 738) = 29.94, *p* < 0.001, η^2^ = 0.0388, but was no main effect of Protagonist, *F*(2, 738) = 1.63, *p* = 0.197, η^2^ = 0.0043, or a significant Knowledge x Protagonist interaction term, *F*(2, 738) = 0.28, *p =* 0.758, η^2^ = 0.00075.

**Meta-Analysis – Raw Data**

**Experiments 1, 2 and 3**

The meta-analytic summary of Experiments 1, 2 and 3 revealed a significant overall Knowledge effect, β (raw effect estimate, logit transformed data) = 13.29; 95% CI = [8.41, 18.61], z = 5.34, *p* < 0.0001. There was no significant effect of Protagonist, β = 0.95; 95% CI = [-3.05, 4.95], z = 0.46, *p* = 0.64, and no significant interaction, β = -5.12; 95% CI = [-11.94, 1.69], z = -1.47, *p* = 0.14. There was no evidence of any significant effects of residual heterogeneity, Q(8) = 9.59, p = 0.30. Overall, the best estimate of the curse of knowledge effect across all conditions was, Cohen’s *d* = 0.394. Individually, the best estimates of the curse of knowledge effect in the in-group, Cohen’s *d* = 0.507 and out-group, Cohen’s *d* = 0.285, conditions.

When the data from Todd et al. [11: Experiment 4] were included, there was again a significant overall Knowledge effect, β = 13.35; 95% CI = [8.80, 17.90], z = 5.75, *p* < 0.0001, and no significant effect of Protagonist, β = 1.1523; 95% CI = [-2.67, 4.97], z = 059, *p* = 0.55. By contrast to our internal meta-analysis, here the interaction term was significant, β = -8.01; 95% CI = [-14.27, -1.74], z = -2.51, *p* = 0.0012. This was driven by a significant curse of knowledge effect across the four experiments in the in-group condition, Cohen’s *d* = 0.513, 95% CI (0.30, 0.72), z = 4.76, *p <* 0.0001, but not the out-group condition, although this approached significance, Cohen’s *d* = 0.195, 95% CI (-0.014, 0.40), z = 1.83, *p* = 0.068. There was evidence of residual heterogeneity, Q(12) = 43.32, *p* < 0.0001, which justified the selection of random effects models.
